# Recurring SARS-CoV-2 variants: an update on post-pandemic, co-infections and immune response

**DOI:** 10.7150/ntno.91910

**Published:** 2024-02-25

**Authors:** Ashmit Verma, Anjali Manojkumar, Anupam Dhasmana, Manish K. Tripathi, Meena Jaggi, Subhash C. Chauhan, Deepak S. Chauhan, Murali M. Yallapu

**Affiliations:** 1 Divyasampark iHub Roorkee for Devices Materials and Technology Foundation, Indian Institute of Technology Roorkee, Uttarakhand, 247667, India.; 2 Samrat Ashok Technological Institute, Vidisha, Madhya Pradesh, 464001, India.; 3 Department of Immunology and Microbiology, School of Medicine, University of Texas Rio Grande Valley, McAllen, Texas 78504, USA.; 4 Department of Biology, College of Science, University of Texas Rio Grande Valley, McAllen, Texas 78504, USA.; 5 Faculté de Pharmacie, Université de Montréal, Montréal H3C 3J7, QC, Canada.; 6 South Texas Center of Excellence in Cancer Research, School of Medicine, University of Texas Rio Grande Valley, McAllen, Texas 78504, USA.; 7 Department of Microbiology and Immunology, Dalhousie University, Halifax, NS, Canada.; 8 Department of Pediatrics, IWK Research Center, Halifax, NS, Canada.

## Abstract

The post-pandemic era following the global spread of the SARS-CoV-2 virus has brought about persistent concerns regarding recurring coinfections. While significant strides in genome mapping, diagnostics, and vaccine development have controlled the pandemic and reduced fatalities, ongoing virus mutations necessitate a deeper exploration of the interplay between SARS-CoV-2 mutations and the host's immune response. Various vaccines, including RNA-based ones like Pfizer and Moderna, viral vector vaccines like Johnson & Johnson and AstraZeneca, and protein subunit vaccines like Novavax, have played critical roles in mitigating the impact of COVID-19. Understanding their strengths and limitations is crucial for tailoring future vaccines to specific variants and individual needs. The intricate relationship between SARS-CoV-2 mutations and the immune response remains a focus of intense research, providing insights into personalized treatment strategies and long-term effects like long-COVID.

This article offers an overview of the post-pandemic landscape, highlighting emerging variants, summarizing vaccine platforms, and delving into immunological responses and the phenomenon of long-COVID. By presenting clinical findings, it aims to contribute to the ongoing understanding of COVID-19's progression in the aftermath of the pandemic.

## 1. Introduction

Coronavirus disease 2019 (COVID-19) is a respiratory disease caused by a novel virus known as severe acute respiratory syndrome coronavirus-2 (SARS-CoV-2). The COVID-19 pandemic has affected millions of people worldwide, and while many have recovered from the acute illness, there is growing concern about the long-term impact on health. Studies have shown that a significant number of individuals continue to experience symptoms even after recovery from COVID-19.

Recent studies have also shown that since the disease primarily affects the respiratory system, there is growing evidence of its potential to cause co-infections with other diseases. Co-infections are defined as the presence of two or more pathogens in the same host at the same time. COVID-19 co-infection with other respiratory pathogens, such as influenza and pneumonia, has been reported 1,2. A study conducted in Wuhan, China, found that 19% of COVID-19 patients had co-infection with another respiratory virus 3. Additionally, COVID-19 patients with co-infection have been shown to have a higher risk of severe illness and death 4. COVID-19 can also lead to co-infection with other non-respiratory diseases, such as cardiovascular disease, diabetes, and cancer. A study conducted in Italy found that 8.3% of COVID-19 patients had a history of cancer, and COVID-19 was more severe in these patients 5. Another study found that COVID-19 patients with diabetes had a higher risk of severe illness and death 6. These findings suggest that COVID-19 co-infection with other diseases may result in worse outcomes and a need for more specialized care.

The diagnosis and treatment of COVID-19 co-infection with other diseases present unique challenges. Co-infection with other respiratory viruses may complicate the diagnosis of COVID-19, as the symptoms may be similar. It is crucial to consider the possibility of co-infection when treating COVID-19 patients, as it may require different treatment strategies. Additionally, patients with co-infections may require more specialized care, such as intensive care unit (ICU) management, which can put a strain on healthcare resources. This review will explore the current understanding of recurring SARS-CoV-2 variants and their co-infection with other diseases and its implications for diagnosis, treatment, and management. Also, in this review, we will explore the post-recovery condition after the pandemic and its potential impact on individuals' immunity and healthcare systems.

## 2. Post-COVID-19 conditions and its recovery trends

### 2.1. Overview of the post COVID-19 conditions

Post-COVID-19 conditions, often referred to as long-COVID, chronic-COVID, or post-acute sequelae of SARS-CoV-2 infection (PASC), are a broad category of ongoing symptoms and medical illnesses that some people continue to have even after they have recovered from COVID-19. These might include exhaustion, shortness of breath, chest discomfort, brain fog, sadness, anxiety, sleep difficulties, and others. These symptoms can also have an impact on other physiological systems. While additional study is done on this newly developing illness, the precise description of post-COVID-19 symptoms is continually changing.

The World Health Organization (WHO) defines long COVID as a range of symptoms that persist for more than 12 weeks and cannot be explained by an alternative diagnosis 7. Several organ systems may be affected, and the symptoms may be recent or persistent. WHO recognises that the scope and definition of long-COVID are still being studied and may change over time.

In a study published in Nature Medicine in February 2021 8, researchers defined long COVID as the persistence of symptoms for more than 28 days after a positive COVID-19 test. The study found that 33% of individuals with mild COVID-19 and 60% of those with severe COVID-19 had at least one persistent symptom for 6 months after infection.

Another study published in The Lancet in March 2021 defined post-COVID-19 conditions as symptoms lasting for at least 12 weeks after the onset of acute COVID-19 illness 9,10. The study identified several common symptoms, including fatigue, shortness of breath, chest pain, joint pain, and depression.

The different problems of post-COVID-19 conditions is still evolving as more and more research is being conducted on this emerging condition. However, the common feature across different definitions is the persistence of symptoms after COVID-19 recovery.

### 2.2. Prevalence of Post-COVID-19 Conditions

Several studies have been conducted to estimate the prevalence of post-COVID conditions. Some of these studies are listed below **(Table 1).**

A study conducted by Carfì et al. in Italy reported that 87.4% of individuals who had been hospitalized for COVID-19 experienced at least one symptom two months after discharge 11. The most common symptoms reported were fatigue, dyspnoea, joint pain, and chest pain.

In a survey of 1,567 individuals with confirmed or suspected COVID-19 in the United States, Davis et al. found that 33% reported not having returned to their usual state of health within 14-21 days after testing 12. Among those with ongoing symptoms, the most commonly reported symptoms were fatigue, cough, and body aches.

Another study by Tenforde et al. reported that among 292 outpatients with COVID-19 in the United States, 35% had not returned to their usual state of health two to three weeks after testing. Among those with ongoing symptoms, the most commonly reported symptoms were fatigue, cough, and shortness of breath 13.

A study by Huang et al. in China reported that among 1,733 hospitalized patients with COVID-19, 76% had at least one symptom at six months after discharge 8. The most common symptoms reported were fatigue or muscle weakness, sleep difficulties, and anxiety or depression.

A study by Bliddal et al. in Denmark reported that among 9,819 individuals who had been tested for COVID-19, 34% reported at least one symptom 60 days after their first positive test 14. The most commonly reported symptoms were fatigue, loss of taste or smell, and difficulty in attention.

The prevalence of post-COVID-19 conditions varies widely depending on the population studied and the definition used. Hospitalized individuals and those with severe cases of COVID-19 appear to be at higher risk for post-COVID conditions. However, even individuals with mild cases of COVID-19 may experience persistent symptoms and health issues after recovery.

### 2.3. Post-COVID rehabilitation

The virus has infected millions of people worldwide and caused significant morbidity and mortality. While some people recover fully after being infected, others experience persistent symptoms, which can significantly affect their quality of life. Research published in the Journal of the American Medical Association (JAMA) in the United States found that 45% of COVID-19 patients who were hospitalised required continuous medical care after release, including rehabilitation services 11.

Rehabilitation programmes for COVID-19 patients have been created all around the world to treat these symptoms. In the United States, for example, the Centres for Disease Control and Prevention (CDC) has created recommendations for COVID-19 patients' rehabilitation that involve a multidisciplinary approach comprising physical therapists, occupational therapists, and speech therapists 15. Several countries, including the United Kingdom, Australia, and Canada, have created similar principles.

The National Health Service (NHS) in the United Kingdom has also established a COVID-19 rehabilitation programme to assist patients in recovering from the virus's effects. A multidisciplinary approach is used in the programme, which includes physiotherapy, occupational therapy, and psychological assistance. According to research published in the British Journal of General Practice, 60% of patients hospitalised with COVID-19 reported continuing experience symptoms three months after release 16.

The Victorian Department of Health and Human Services in Australia has also established a COVID-19 rehabilitation programme to assist patients in recovering from the virus's effects. A variety of therapies are included in the programme, including physiotherapy, occupational therapy, speech therapy, and psychological support. According to a research published in the Medical Journal of Australia, 64% of COVID-19 hospitalised patients had persistent symptoms even six months after release 17.

### 2.4. Clinical conditions amid to post-COVID

**Figure 1:**
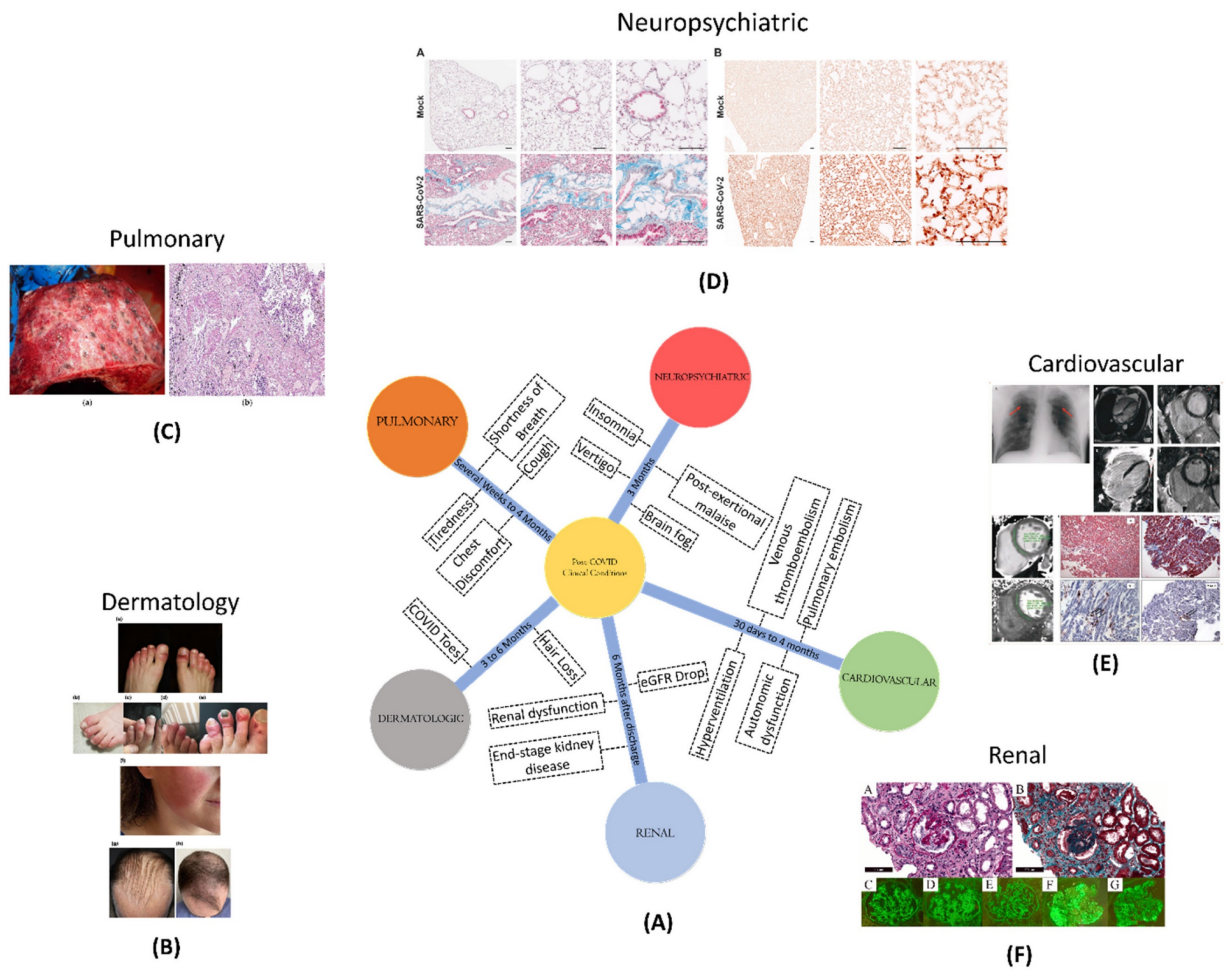
** (A)** A schematic showing different clinical conditions and their symptoms, amidst to COVID-19 disease; **(B)** Dermatological manifestation of COVID-19 such as COVID toe, hair loss; Adopted with permission from ref. 21; **(C)** Macroscopic and microscopic aspect of the lung in a 55-year-old male patient with COVID-19; adopted from ref^22^; **(D)** Masson's trichrome staining of paraffin-embedded lung sections of K18-hACE2 marker and Immunohistochemical staining of B1R (brown) in lung tissue of recovered from mild SARS-CoV-2 infection to analyse the post-COVID neuropsychological disorders in mice models; adopted from ref 23; **(E)** number and types of thrombotic events such as venous thromboembolism, pulmonary embolism, stroke, hyperventilation; adopted from ref 24; **(F)** risk and excess burden of post-acute COVID-19 kidney histomorphological and immunofluorescence features; adopted from ref 25.

In the midst of the post-COVID scenario, different clinical conditions have been observed across various organ systems (**Figure 1**). Pulmonary conditions, such as shortness of breath, cough, and fatigue, can extend for several weeks to 4 months. Cardiovascular conditions, such as inflammation and blood clotting, pose risks between 30 days and 4 months post-diagnosis. Neuropsychiatric conditions, affecting cognition, memory, and sensory functions, can persist for over three months. Renal complications, such as an increased risk of acute damage and persistent dysfunction, pose risks up to six months post-discharge. Dermatologic issues, like COVID toes and hair loss, may persist from three to six months post-discharge.

#### 2.4.1. Pulmonary conditions

In post-COVID-19 patients, pulmonary problems might include a variety of respiratory symptoms such as shortness of breath, cough, chest discomfort, and tiredness. These symptoms may last for several weeks or even months, and in rare cases, they may result in long-term lung damage as well 18.

Pulmonary fibrosis is one of the most serious pulmonary diseases in post-COVID-19 patients. It is a form of lung illness that arises when lung tissue gets damaged and scarred, making proper lung functioning difficult. According to a research report, those who have recovered from COVID-19 may be at a higher risk of acquiring pulmonary fibrosis 19.

Chronic obstructive pulmonary disease (COPD) is another pulmonary ailment seen in post-COVID-19 patients. COPD is a chronic lung condition that causes breathlessness, coughing, and wheezing. Those with a history of COVID-19 infection may be at a higher risk of getting COPD, according to research 20.

#### 2.4.2. Cardiovascular conditions

Different studies conducted on both the hospitalized and non-hospitalized patients shows high chances of major adverse cardiovascular events between a time period of 30 days to 4 months post-COVID-19 diagnosis 26,27. The studies were verified by using different diagnostic testing techniques, such as electrocardiography, echocardiography, cardiac magnetic resonance (CMR), and cardiopulmonary testing. These trials had varying outcomes, most likely owing to variation in patient population. A review of electrocardiographic data shows that dynamic alterations (e.g., depolarization and repolarization abnormalities, as well as the prevalence of arrhythmias) during acute illness tend to disappear in the majority of hospitalised patients within 6 months. Autonomic dysfunction, such as postural orthostatic tachycardia syndrome, neurocardiogenic syncope, and orthostatic hypotension, is becoming more common in addition to inappropriate sinus tachycardia 28.

The results of CMR investigations have received a lot of interest from both the medical and lay sectors. While early research outcomes revealed disturbingly high rates (60%) of chronic cardiac inflammation 2-3 months after infection, subsequent investigations revealed a much lower prevalence (40%) 29. These research' intrinsic limitations, such as individuals' comorbidities and the lack of a comparator group, were recognised and discussed further 30. While other investigations, including one in healthcare professionals, have now identified a decreased frequency of CMR related abnormalities, with no significant difference in 6-month CMR results between seropositive and seronegative people 31.

Abnormalities in echocardiography, such as the enlargement and malfunction of the right ventricle in the initial stages of COVID-19, have been found to improve in most patients 32. However, due to the lack of pre-COVID-19 assessments, the interpretation of these findings is often limited.

In the context of comprehending the physiological changes following COVID-19, Cardiopulmonary exercise testing has been useful in comprehending the physiological changes following COVID-19, which includes reduced oxygen consumption due to impaired oxygen extraction, hyperventilation or dysfunctional breathing. Several factors like inadequate nourishment, medication use (e.g., dexamethasone), mechanical ventilation, and deconditioning may influence these outcomes 33-35.

The cardiac problems related to COVID-19 also involve blood clotting such as pulmonary embolism and venous thromboembolism. It is speculated that the increased risk of blood clots following COVID-19 is linked to endothelial cell activation and a hyperinflammatory state with unknown duration. Studies indicate that the occurrence of venous thromboembolism is less than 5%, but due to the uncertainty surrounding the duration and risk of these complications following acute COVID-19, standard risk assessment tools are recommended 36.

#### 2.4.3. Neuropsychiatric

Neuropsychiatric issues can have a significant impact on a person's quality of life even in non- hospitalized COVID-19 patients. Symptoms such as cognitive or memory problems, commonly known as brain fog, vertigo, post-exertional malaise, insomnia or other sleep issues, headache, and taste or smell problems persist for over three months after diagnosis 37. The data on each of these symptoms is not precise due to the varied questionnaires and surveys used in various studies. Nonetheless, there is a higher incidence of long-lasting symptoms among women than men, as demonstrated in a prospective study of non-hospitalized patients who visited a neuro-COVID-19 clinic, where 70% of patients with neurologic symptoms lasting over six weeks were female 38.

The importance of these long-term neurological disorders is indicated by the findings of an international online study, which discovered that around 30% of participants aged 30-59 who had cognitive impairments also had a diminished capacity to operate at work 37. This percentage is thought to be an underestimate among minority populations e.g., Black, Latino, Indigenous, and others, who are more prone to develop SARS-CoV-2 but are underrepresented in research and post-COVID-19 hospitalisation treatment 39,40. Hyposmia and anosmia are further neurological symptoms that may be induced by olfactory sensory neuron loss. Despite this, recent research found that recovery from these symptoms is often excellent, with nearly full recovery occurring within a year 41.

Numerous initiatives have been launched to promote further research in this area, including the European Academy of Neurology's NeuroCOVID-19 task force and the United States-based National Institute of Neurological Disorders and Stroke NeuroCOVID Project 42. These groups aim to consolidate and focus efforts to understand the long-term effects of COVID-19.

#### 2.4.4. Renal

COVID-19 is linked to an increased risk of acute renal damage and severe adverse kidney consequences such as a decrease in estimated glomerular filtration rate (eGFR), end-stage kidney disease, or all-cause mortality. A cohort study of over 90,000 veterans with COVID-19 found that 30-day survivors had a greater drop in eGFR than controls 43. Approximately one-third of hospitalized COVID-19 survivors have persistent renal dysfunction six months after discharge from hospital, and a subset of these patients still requiring dialysis, according to few preliminary studies 8,44.

#### 2.4.5. Dermatologic

The most common dermatological manifestations of acute COVID-19 have been acral chilblain-like or pernio-like lesions, also known as COVID toes 45. While these lesions typically resolve within two weeks, a subset of patients in multidisciplinary or specialty clinics have been observed to have persistent chilblain lesions. Screening for underlying causes is recommended in such cases, and the use of nailfold capillaroscopy has been suggested for identifying potential microcirculatory morphological alterations, as is used in rheumatologic practice 45-47. Early reports also suggest the possibility of rheumatologic illnesses, such as inflammatory arthritis and large-vessel vasculitis, following the resolution of acute infection. However, more research is needed to understand the relationship between COVID-19 and these dermatologic and rheumatologic manifestations 46,47.

Although earlier research suggested that around 20-22% of patients experienced hair loss 3-6 months after recovering from COVID-19, the exact relationship between hair loss and COVID-19 remains unclear and requires further investigation 8,48. Recent studies, including a large-scale Korean national cohort study involving over 226,000 individuals who tested positive for SARS-CoV-2, have found no significant association between COVID-19 and the development of alopecia areata compared to control groups, with an incidence rate ratio of 0.6 49.

### 2.5. Advantages and disadvantages of the post-COVID situation

The COVID-19 pandemic with its unprecedented reach and impact, has undeniably altered the global landscape. We now find ourselves traversing the uncharted territory of the post-COVID era, grappling with its complexities and uncertainties. The post-pandemic situation has brought both new opportunities and challenges to address, demanding a nuanced understanding of both its advantages and disadvantages.

#### 2.5.1. The emergent advantages

The pandemic has exposed many vulnerabilities within global health systems, pushing a significant shift in priorities. Increased investments in research, infrastructure, and pandemic preparedness initiatives are now making the way for improved diagnostics, vaccines, and treatment strategies. This pre-emptive strategy stands to equip us better for future outbreaks, potentially mitigating their impact. A rapid technological outbreak had been seen driven by the urgent need for social distancing and remote work. Technological solutions like, telehealth, remote work platforms, and digital learning solutions emerged as essential tools, offering greater accessibility, flexibility, and potentially improving healthcare delivery, work-life balance, and educational opportunities 50-52. A newfound appreciation for basic necessities like health, food security, and social connections has emerged. This shift, if sustained, can contribute to a more mindful and values-driven society, prioritizing well-being over excessive consumerism. Finally, the pandemic has increased the emphasis of hygiene and sanitation. Now, people are more aware of their surrounding and following hygiene protocols in public spaces.

#### 2.5.2. The lingering challenges

A substantial portion of the population continues to grapple with the long-term health effects of COVID-19, known as long-COVID. This diverse range of symptoms, from fatigue and brain fog to organ damage, poses a significant burden on individuals and healthcare systems alike 9. Ongoing research and dedicated support systems are crucial for effective management and improved quality of life for those affected.

The pandemic had a more significant impact on vulnerable communities, making existing social and economic inequalities worse. Increased poverty, unemployment, and food insecurity continue to plague marginalized populations. To address these disparities, targeted interventions and policies are essential to ensure equitable access to resources and opportunities 53.

The isolating and anxiety-inducing nature of the pandemic has contributed to a surge in mental health challenges like depression and anxiety. Increased access to mental health services and resources is critical to support individuals struggling with these issues and foster a culture of emotional well-being 38.

School closures and economic lockdowns disrupted education and employment opportunities, particularly for marginalized communities. This necessitates innovative approaches to ensure equitable access to education and economic opportunities in the post-COVID era 54.

As we navigate the post-COVID landscape, it is vital to acknowledge both the challenges and opportunities that lie before us. By fostering continued research, collaboration, and addressing existing inequalities, we can build a more resilient and equitable future. We must leverage the positive changes and advancements while actively addressing the lingering difficulties to create a world where health, well-being, and opportunity are accessible to all.

## 3. Variants and their mutations

**Figure 2:**
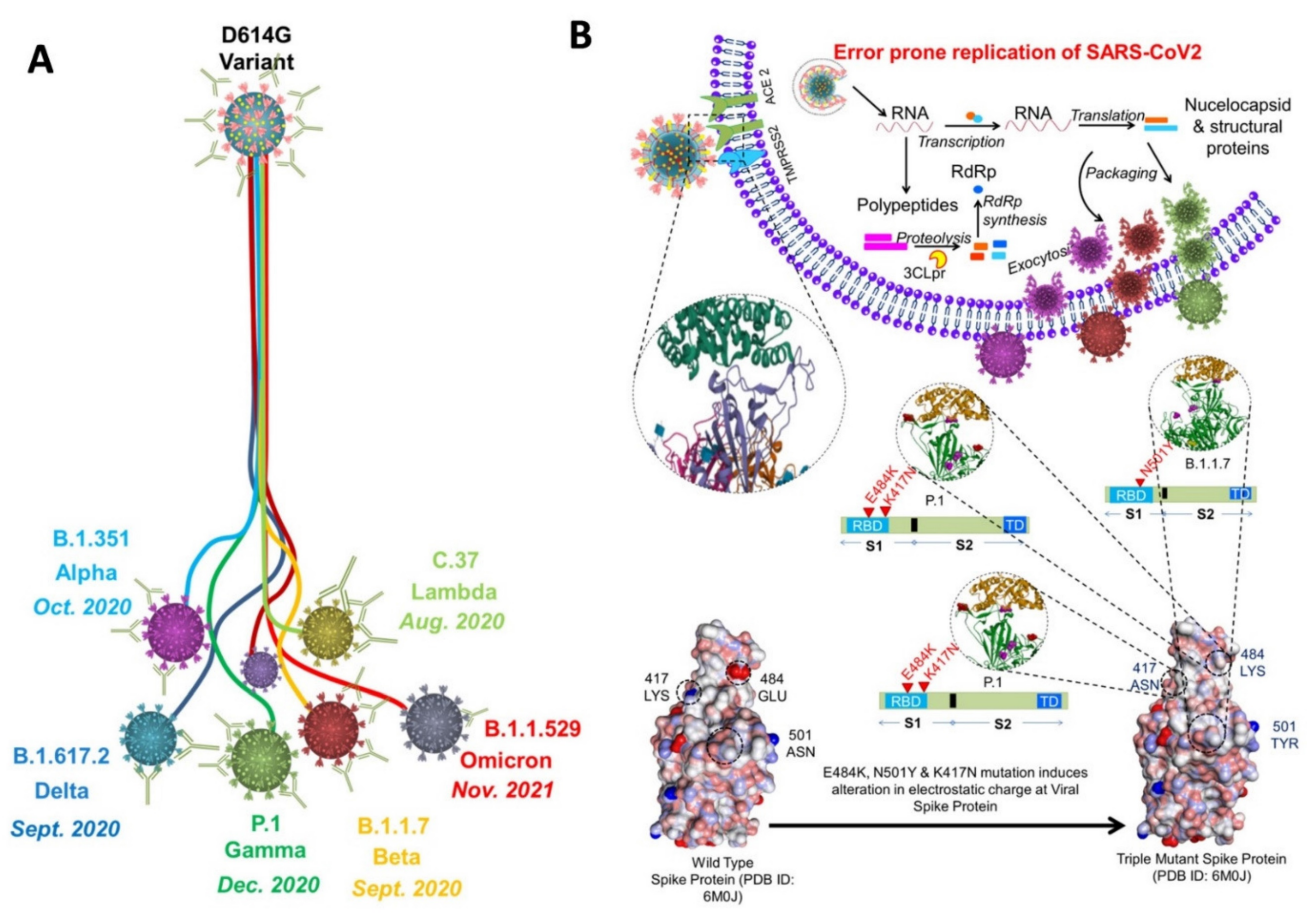
** A)** Schematic showing the development of variants of concern from the SARS-CoV-2 (D614G variant). The 614G is the earliest spike protein mutant of SARS-CoV-2 which swiftly dominated the 614D (wild type) all over the world. **B)** Physiology of SARS-CoV-2 variants showing error prone replication. The error prone replication of SARS-CoV-2 helps to produce most suitable variants by spontaneous mutation on the receptor binding domain (RBD) of the spike protein. These mutations change the infectivity index of the variants. In the bottom, the spike protein of the wild type is compared with the spike protein of the triple mutant, wherein 417 LYS has been replaced by 417 ASN; 484 GLU replaced by 484 LYS; and 501 ASN replaced by 501 TYR. All these mutations are at the RBD, and known to alter the electrostatic charge which effect the infectivity index of the variants 55-58.

Since the emergence of SARS-CoV-2 in 2020, it continues to spread around the globe by developing new variants **(Figure 2A)**. Each mutation produces a slightly different strain of SARS-CoV-2. According to WHO, at present, there are almost twelve variants of SARS-CoV-2 that has been recorded worldwide. These are named as Alpha, Beta, Gamma, Delta, Delta plus, Epsilon, Eta, Theta, Iota, Kappa, Lambda, and Omicron.

Out of all these SARS-CoV-2 variants, five variants are considered to be variants of concern that has spread rapidly throughout the world. These variants include Alpha (B.1.1.7), Beta (B. 1.351), Gamma (P.1), Delta (B.1.617.2), and Omicron (B.1.1.529) 55-58. To understand the variants, we must first understand the binding phenomenon of SARS-CoV-2 59. In SARS-CoV-2, 5 key parts are responsible for binding: The RBD, N terminal domain, connector domain, central helix, and the transmembrane. SARS-CoV-2 uses the homotrimeric protein or spike protein for binding to the ACE2 receptor via RBD. As binding stabilized, membrane of host cell and virus fuse together, and then the replication of the virus can go underway. Each mutation of SARS-CoV-2 can further change or enhance this process (**Figure 2B**).

**Figure 3:**
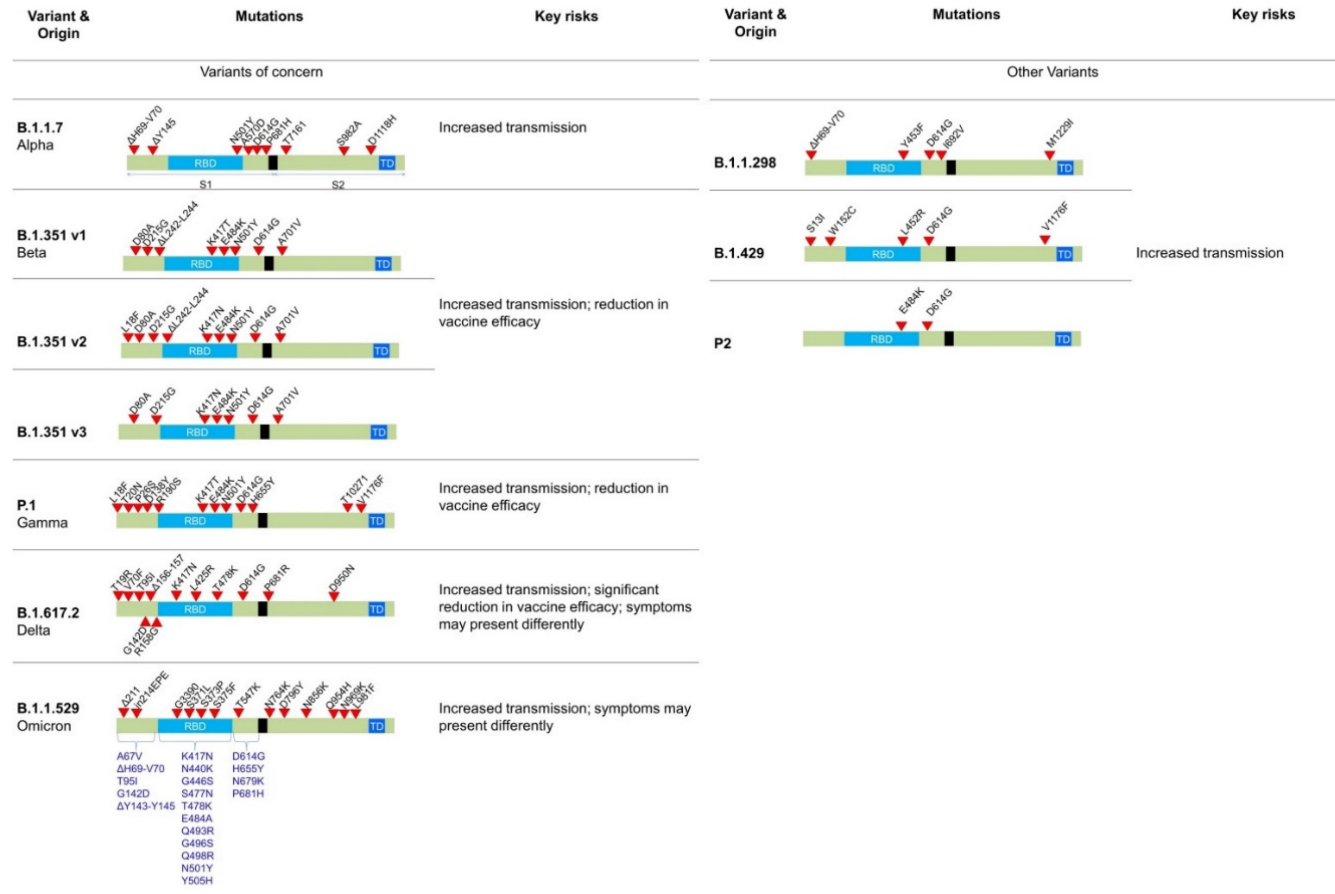
Mutations and characteristic features of SARS-CoV-2 major variants. In the case of B.1.1.529 (Omicron), mutations listed in blue color already have been reported also in other variants of SARS-CoV-2 virus.

A number of mutations have been identified in different variants of SARS-CoV-2, which has been illustrated in **Figure 3**. For example, the 69del, 70del, 144del, (E484K*), (S494P*), N501Y, A570D, D614G, P681H, T716I, S982A,'D1118H (K1191N*) mutations is Alpha variant, D80A, D215G, 241del, 242del, 243del, K417N, E484K, N501Y, D614G, and A701V in Beta variant, etc. Out of these Omicron variant has the maximum number of mutations (around 15 in RBD and 30 in total).

### 3.1. Alpha

**Figure 4:**
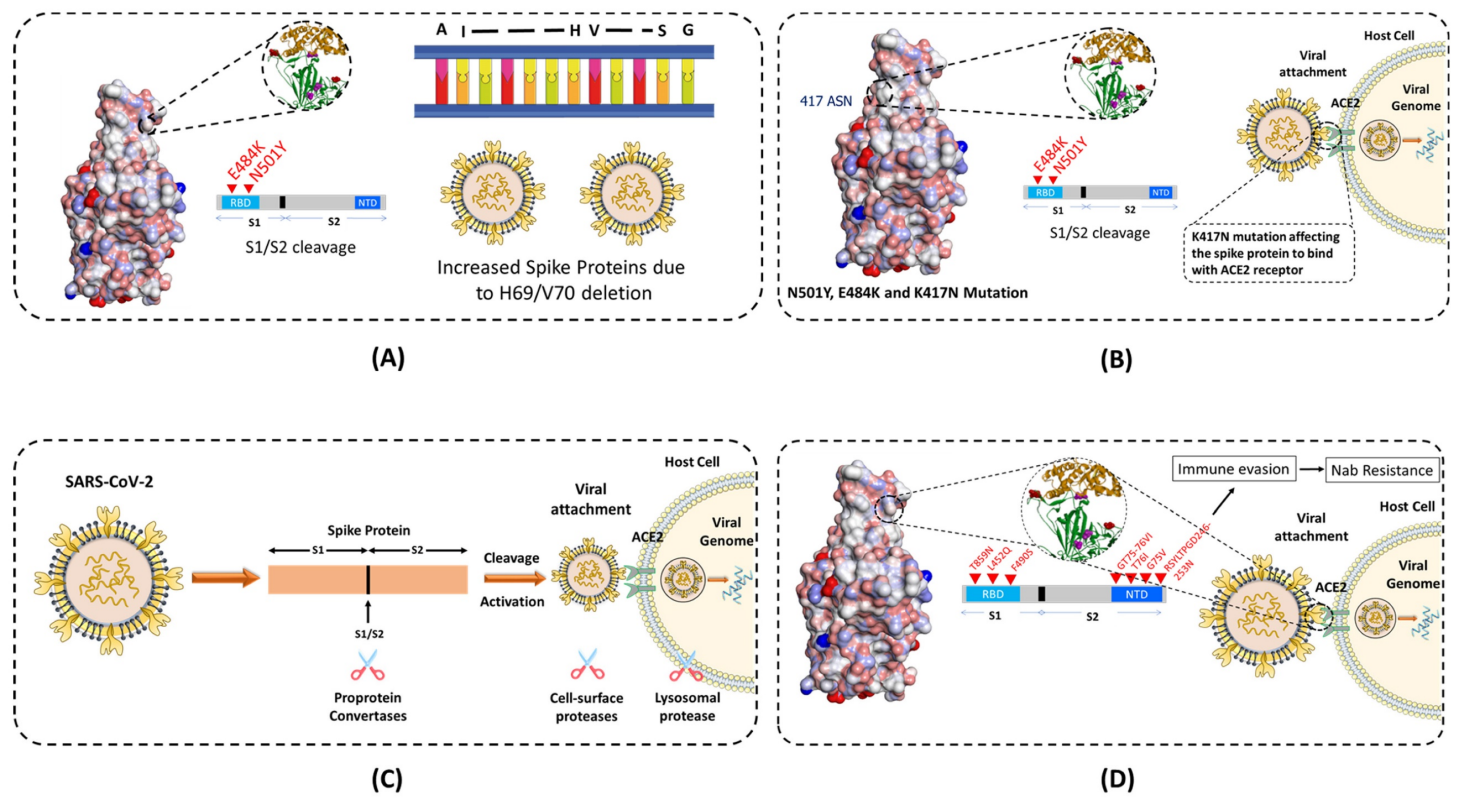
Schematics showing how different mutations near the S1 and S2 furin cleavage site, which virus uses to enter and bind to host cells are contributing in transmissibility and infection rate across different variants of the SARS-CoV-2. **(A)** E484K and N501Y mutation at the RBD and deletion of H69 and V70 amino acids in Alpha variant, which allows the virus to evade the vaccine and infect both vaccinated and non-vaccinated patients. **(B)** and **(C)** K417N mutation in variants, affecting the spike protein to bind with ACE2 receptor. **(D)** Different other mutations in other variants to invade the host cell.

The Alpha (B.1.1.7) variant is also known as the United Kingdom (UK) variant [10-12]. It has spread to more than 94 countries and is 35 - 45% more infectious than the original COVID-19 virus. It is well known that the COVID-19 virus relies heavily on the binding of spike protein to the ACE2 receptor for infecting human cells. The Alpha variant contains many variations to the spike protein **(Figure 3)**. It allows the spike protein to bind to human cells much easier than the original strain. The Alpha variant consists of 17 mutations (Key mutation: N501Y) and 3 deletions 60,61. Specifically, 2 amino acids- H69 and V70 are completely deleted in the spike protein. This deletion doesn't affect the efficacy of neutralizing antibodies (nABs) found after vaccination or post-infection. However, H69 and V70 deletion does increase the number of mature spike proteins on the virus's surface 62. The other mutations of the Alpha variant include 69del, 70del, 144del, (E484K*), (S494P*), N501Y, A570D, D614G, P681H, T716I, S982A,'D1118H (K1191N*). The asterisk denoted mutations haven't been observed in all copies of alpha variants. The P681H mutation occurs near the S1/S2 furin cleavage site, which virus uses to enter and bind to host cells **(Figure 4 A)**. This mutation allows the virus to evade vaccine and infect both vaccinated and non-vaccinated patients 63,64. Although, those who have received the COVID-19 vaccine shows less severe symptoms than those who haven't. Also, there is no impact on monoclonal antibody treatments.

### 3.2. Beta

The Beta (B.1.351) variant, also known as the South African variant, is a fast-spreading variant of SARS-CoV-2. Till now, Beta variant has been detected in more than 60 countries due to 50% increase in transmission than the original strain. This variant has three key mutations; N501Y, E484K, and K417N 65. The spike protein mutations include D80A, D215G, 241del, 242del, 243del, K417N, E484K, N501Y, D614G, and A701V. Similar to the Alpha variant, N501Y mutation is located in the RBD of the spike protein **(Figure 3)**. It allows the virus to become more transmissible by facilitating the binding to the ACE2 receptor. The 501 mutation is the only mutation that occurs within the ACE2 patch. Also, Beta variant easily passes the natural immune defences via the E484K mutation **(Figure 4 C)**. This mutation has also proven to be less neutralized against serum antibody therapy. The last key mutation of the Beta variant is the K417N mutation. While there is little information on the K417N mutation, it is known that it is similar to the N501Y mutation. Likewise, it affects the spike protein and allows it to easily bind the ACE2 receptor. Further, when K417N mutation is in tandem with N501Y, Beta variant can not only gain access to the cells faster, but also able to reduce the efficacy of the nAbs 66,67. Also, it significantly reduces the efficacy of bamlanivimab and etesevimab (monoclonal antibody treatment) combination, but other emergency use authorization (EUA) monoclonal antibody treatments are still available 68,69. The reduced neutralization of convalescent and post-vaccination sera has also been experienced.

### 3.3. Gamma

The Gamma variant has a number of spike protein mutations **(Figure 3)**, including L18F, T20N, P26S, D138Y, R190S, K417T, E484K, N501Y, D614G, H655Y, and T1027I 70. In these mutations, the most dangerous are N501Y, E484K, and K417T. Similar to the mutations of the Beta variant, Gamma variant is also able to bind to the ACE2 receptors easily and evade the body's immune system via N501Y and E484K, respectively **(Figure 4 C)**. Additionally, K417T and E484K mutations allow increasing the affinity of the virus to the ACE2 receptor. This leads to a stronger binding to the host cell, and thus a higher rate of infection and transmission. Also, these mutations significantly reduce susceptibility to the combination of bamlanivimab and etesevimab monoclonal antibody treatment, but other EUA mono-clonal antibody treatments are effective 68,69. Also, it reduces the neutralization by convalescent and post-vaccination sera 71. The gamma variant has proven to be 2.5 times more contagious in comparison to the original strain. However, no data support that this strain is also more deadly than the original strain.

### 3.4. Delta

While the 3 strains of the SARS-CoV-2 were being closely monitored, the 4th and most worrisome Delta variant emerged in India. The Delta variant is said to be 2x more contagious than the other strains 72. Delta-Plus (AY.1 and B.1.617.2.1) is a sub-variant of the Delta variant (B.1.617.2). The signature spike mutations in the Delta and Delta-Plus variant includes T19R, (V70F*), T95I, G142D, E156-, F157-, R158G, (A222V*), (W258L*), (K417N*), L452R, T478K, D614G, P681R, and D950N 61 **(Figure 3)**. The asterisk denoted mutations haven't been observed in all copies of Delta variants. One of the key mutations in the Delta variant is K417N which is alarming because it is located to the RBD of the spike protein, allowing far easier binding to the host cell 73. The Delta variant also has 2 mutations (P681R and L452R) that aren't seen in any other strain. The P681R mutation is located in the furin cleavage site and aids in the binding of the spike protein to healthy human cells by enhancing spike fusogenic activity **(Figure 4 A)**. As stated earlier, cell to cell fusion is a key to the stability and replication of the virus within healthy cells. All these mutations make the Delta variant 50% more transmissible than the Alpha variant 74. Along with P681R mutation, L452R has also been a major cause for concern. The L452R mutation helps in increasing the stability of S protein by creating a pathway to latch the virus onto the host cell. It also assists in injecting down the genetic material into the host cell. The more stable the spike protein, the more likely viral replication can occur. The L452R mutation also allows the impairment of the nAbs. After nAbs inactivation, the host is unable to defend itself and, therefore, more likely to become sick or even hospitalized. L452R is an alarming mutation because some vaccines are effective purely by their ability to create only Abs 75. Studies have shown that no vaccine is 100% effective against the Delta variant, though the Pfizer vaccine claims up to 95% efficacy against hospitalization if fully vaccinated 76. These key mutations also affected the other treatments of COVID-19, along with less susceptibility against Bamlanivimab.

### 3.5. Lamda

Lambda variant is considered one level below the other variants of concern. The Lambda variant, also known as C.37, has many variations on the spike protein. These mutations include G75V, T761, Δ246-252, L452Q, F490S, D614G, and T859N **(Figure 3)**. These mutations allow spike proteins to bind to the ACE2 receptors with more strength than the original virus. As a result, the infectivity rate of this variant is higher than the original virus strain **(Figure 4 D)**. It is important to note here that the L452Q mutation is very similar to the L452R mutation discussed in the Delta variant, which makes this virus also more infectious. Additionally, the F490S mutation has been associated with resistance to previously acquired Abs 77. Also, there is a 7 amino acid deletion in the N domain of the spike protein, known as RSYLTPGD246-253N mutation. It also gives the lambda variant an ability to doge previously acquired Abs 78,79.

### 3.6. Omicron

As of November 26th, 2021, the Omicron variant has been classified as a variant of concern by the World Health Organization (WHO). According to WHO, little is known about the Omicron variant besides the fact that it has already reached more than 50 countries with very high number of cases 72,80. The Omicron virus has 30 known mutations leading to changes in the spike sequence. 15 of the mutations are located in the RBD of the virus **(Figure 3)**. The Omicron virus is thought to have increased infectivity as some of the mutations identified in the S1-S2 cleavage site are similar to the mutations found in the Delta and Alpha variants. The Omicron virus has the highest transmission rate and severity among all the other variants of the SARS-Cov-2 virus **(Figure 4 A)**. To counter the spread of the Omicron variant WHO recommended the use of masks, vaccines, and booster doses. Many monoclonal antibody treatments, such as Bamlanivimab, are thought to be ineffective similar to the Delta variant. Also, detailed studies are needed to examine the efficacy of vaccines against the Omicron variant.

Since 2021, several studies and real-world data have been published that suggest that COVID-19 vaccines can provide some level of protection against the Omicron variant.

A study published in the New England Journal of Medicine in January 2022 analysed data from a cohort of healthcare workers in South Africa and found that the Pfizer-BioNTech vaccine provided 70% protection against symptomatic infection with the Omicron variant 81. However, the protection was lower compared to the protection provided against previous variants. A study from the UK published in The Lancet in February 2022 showed similar results, with the Pfizer-BioNTech vaccine providing 53% protection against symptomatic infection with the Omicron variant 82.

Real-world data from Israel, which has one of the highest vaccination rates in the world, showed that a third booster dose of the Pfizer-BioNTech vaccine significantly enhanced protection against the Omicron variant. Also, a study published in The Lancet in February 2022 showed that the booster dose increased the level of neutralizing antibodies by more than 25-fold, providing more than 90% protection against symptomatic infection with the Omicron variant 83. While, the Johnson & Johnson vaccine has been found to be less effective against the Omicron variant compared to other variants. A study from South Africa published in The Lancet in February 2022 found that the Johnson & Johnson vaccine provided 33% protection against symptomatic infection with the Omicron variant. However, the vaccine still provide some level of protection against severe disease, hospitalization, and death 84.

It's important to note that the situation is rapidly evolving, and new advanced variants of the Omicron variant are being identified all over the place 85*.* A very recent report came from China where the population being infected by a subvariant of Omicron i.e., XBB variant. By October 2023 it is been speculated that more than 99 million total confirmed cases can be recorded from this variant 86. As per the data from WHO, over half a million new cases have been reported during the period of 28 October 2023 to 19 November 2023. Country wise the number of new cases reported were - Russian Federation (121482), Italy (104165), Singapore (64021), Australia (34635), and Poland (21879). The variants of interest (VOIs) currently are: XBB.1.5, XBB.1.16, EG.5 and BA.2.86; and the variants under monitoring (VOMs) are: DV.7, XBB, XBB.1.9.1, XBB.1.9.2 and XBB.2.3. Out all the VOIs and VOMs, EG.5 is found to be the most prevalent one, which has been now reported in 89 countries. With this it is important to note that the rate of testing and sequencing has reduced globally, which has posed challenges to gather data to estimate the severity impact of emerging SARS-CoC-2 variants 87.

## 4. COVID-19 vaccines and vaccines induced immunity

**Figure 5:**
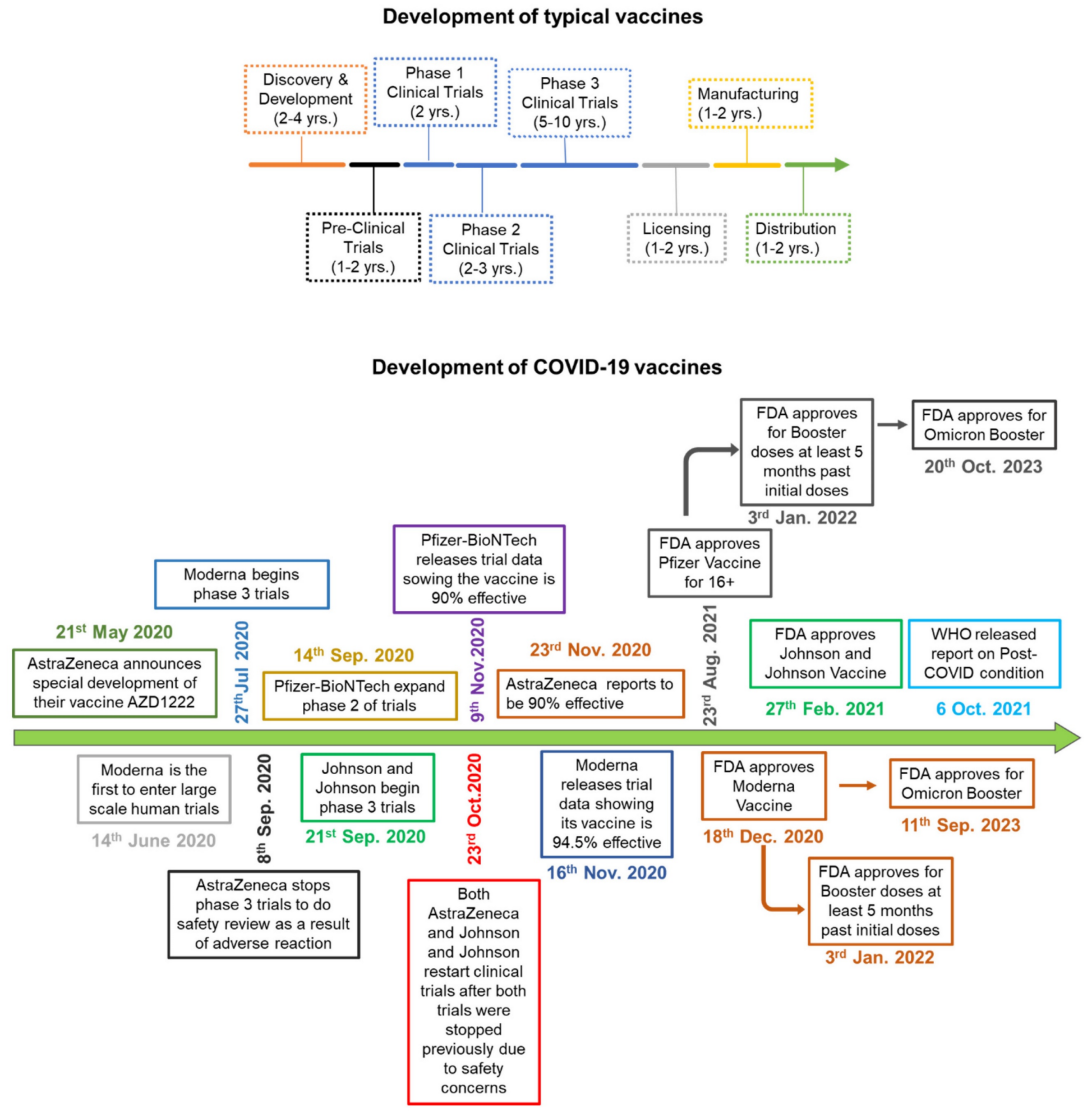
Schematic comparing the process of vaccine development. **A)** Development of typical vaccine: The multicolor arrow indicates the timeline and the amount of time it takes to develop a normal vaccine before COVID-19. **B)** The green arrow indicates how COVID-19 vaccines were developed, and how each phase was overlapped and shortened significantly in comparison to normal vaccine development.

When a new virus threatens the population, the best solution is to develop vaccines. Traditionally speaking, the vaccine is a weakened version of the virus itself. This allows the body to fight the weak strain and make the appropriate Abs. The Abs are then placed on standby until the real virus attacks 88. There are multiple platforms for COVID-19 vaccine, of which RNA-based and viral vector vaccines are most popular. The RNA-based vaccines can be easily prepared because of no usage of fermentation and culture rather than synthetic process 88. Following is the brief account of vaccine development and efficacy in controlling the COVID-19 variants **(Figure 5). Table 2** summarizes various vaccines granted EUA, detailing their approval by countries. For instance, Pfizer-BioNTech has been authorized in 140 countries, including the United States, Switzerland, and Australia. The table provides information on the technology employed, distinguishing between RNA-based, viral vector, protein-based, or inactivated virus vaccines. It also outlines the active ingredients in different vaccines, such as mRNA encoding the spike glycoprotein in the Moderna vaccine and the ChAdOx1-S recombinant vector encoding the spike glycoprotein in the AstraZeneca vaccine. Additional details include storage temperature requirements, dosage specifications, efficiency, advantages and disadvantages, and the primary countries involved in major clinical trials.

### 4.1. RNA vaccines

**Figure 6:**
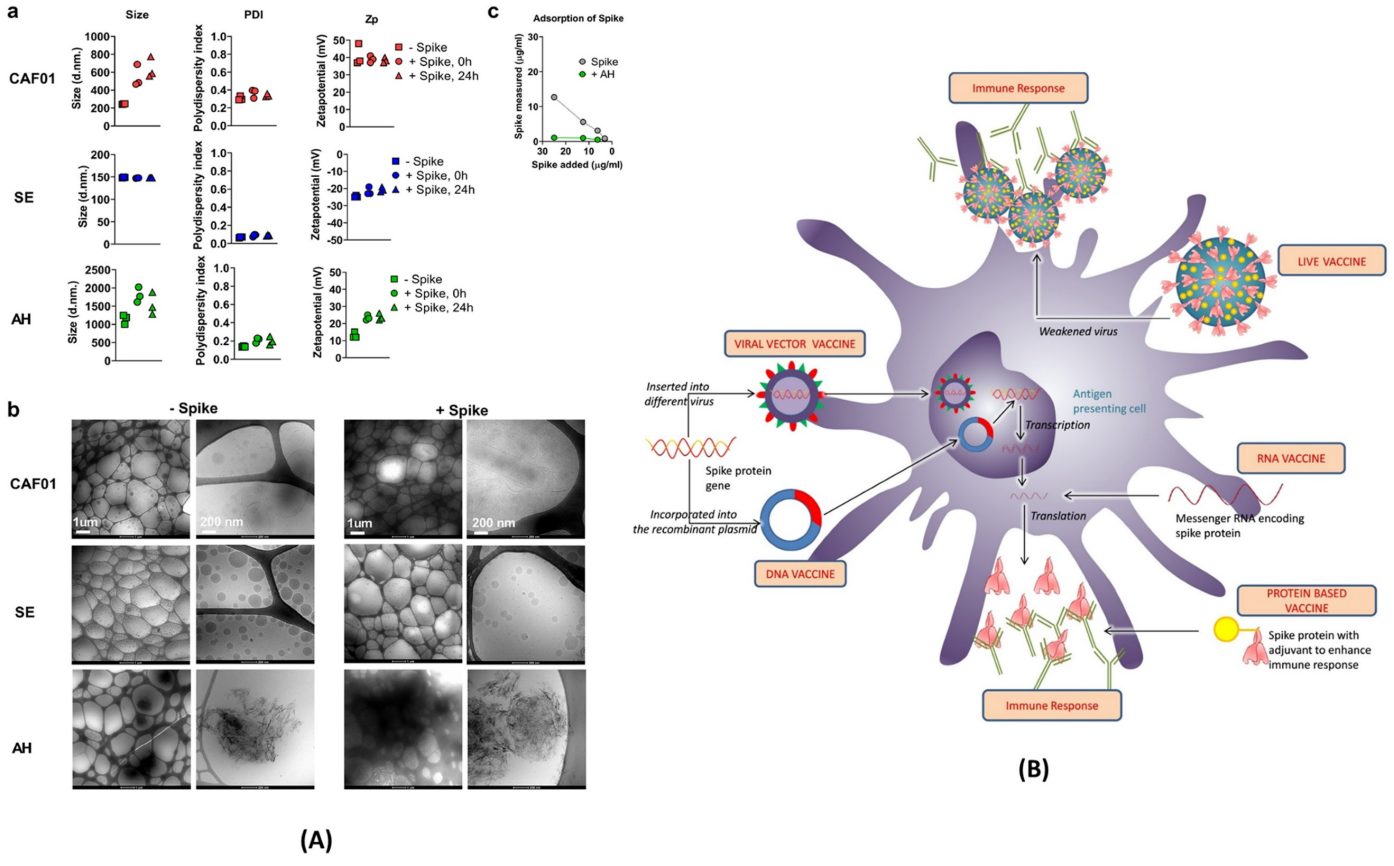
**(A)** Characterization of vaccine formulations. Different adjuvant systems, cationic liposomes (CAF01), squalene emulsion (SE) and aluminium hydroxide (AH), were tested for compatibility with pre-fusion stabilized (S-2P) spike trimer protein. **a)** The particle size comparison, polydispersity index (PDI, middle panels) and the Zeta potential (Zp, right panel). **b)** Adsorption of spike protein to aluminium hydroxide (AH) and the Cryo-TEM micrographs. **c)** Adsorption of the spike protein determined by measuring protein content recovered in the supernatant after ultracentrifugation; adopted with permission from ref 89. **(B)** The strategies used for the development of vaccine for COVID-19. The viral vector and RNA based vaccine are ahead in global outreach, and also available in the form of 3rd and 4th booster shots for SARS-CoV-2 variants; adopted from ref 90.

To counter COVID-19 in a safe and effective way, pharmaceutical companies like Pfizer and Moderna have developed RNA-based vaccines. SARS-CoV-2 enters human cells with the help of spike protein binding to ACE-2 receptor of human cells. Thus, scientists determined the specific portion of RNA that only codes for the spike protein and transformed it into the vaccine. As showcased in **Figure 6 (A) and (B)**, various other vaccine strategies are also being explored. Live attenuated vaccines utilize weakened SARS-CoV-2 strains to induce immunity. Viral vector vaccines employ harmless viruses as carriers to deliver genes encoding the spike protein. Protein subunit vaccines directly introduce the isolated spike protein to stimulate the production of antibodies. Regardless of the chosen strategy, all COVID-19 vaccines share a common goal: to elicit a potent humoral immune response. By promoting the production of antibodies targeting the spike protein, these vaccines effectively neutralize the virus and prevent its entry into host cells, thereby hindering infection. Extensive clinical trials have rigorously evaluated the safety and efficacy of currently available COVID-19 vaccines. These trials have demonstrated the potential of these diverse and innovative vaccines to significantly reduce the incidence of severe illness, hospitalization, and mortality associated with COVID-19. They represent a critical line of defense in the global fight against this ongoing pandemic.

Drew Weissman in his article discussed the improvements in the new mRNA vaccines to increase protein translation, modulate innate and adaptive immunogenicity and improve delivery 91. These spike coding RNAs are not harmful to the body because they don't represent the whole virus. After vaccination, host cells start synthesizing spike proteins which invoke immune cells to develop Abs corresponding to the spike proteins of the virus. When the actual SARS-CoV-2 attacks, immune cells pre-exposed to spike proteins quickly recognize the virus and attack with the already developed Abs.

The different RNA-based vaccines have different efficacies; the Moderna vaccine claimed to be 94.1% efficacious while Pfizer-BioNTech claimed to be 95% in their phase 3 clinical trial 92 93. However, during the clinical trials of the Pfizer-BioNTech vaccine, 84.7% of participants reported at least one reaction at the site of injection 71. Additionally, 77.4% of participants reported having systemic reactions lasting up to 7 days. The frequency of post-vaccination symptoms was higher after the second dose. The symptoms ranged from mild to moderate severity. Similar to Pfizer-BioNTech, participants in the Moderna clinical trials also reported mild to moderate symptoms after receiving the second dose. The symptoms include fatigue, headache, fever, chills, vomiting, diarrhoea, and muscle pain. Further, fifty-eight cases of imbalanced lymphadenopathy were reported among the Pfizer-BioNTech trials. Those who were affected reported swelling in the neck and arm area 2-4 days post-vaccination. The lymphadenopathy was reported to last roughly ten days. In addition, some patients also reported myocarditis and or pericarditis after receiving the mRNA vaccines. Mostly, male adolescents and adults have shown these issues after receiving the second dose of either Pfizer-BioNTech or Moderna vaccine 94. Also, it is important to note that these issues were responsive to medical care.

As the pandemic continued, more and more strains of the SARS-CoV-2 arose, with increasing concern regarding the efficacy of RNA-based vaccines. Some variants, such as Delta and Omicron, have certain mutations to evade vaccines and cause infection. Regardless, it has been found that those who have received the vaccine have higher immunity and faster recovery than unvaccinated people 71.

### 4.2. DNA vaccines

DNA vaccines tackle the same problem in different ways. In the case of DNA vaccine, a fragment of the SARS-CoV-2 DNA, specifically, the S or spike protein, is isolated and placed into a plasmid. This plasmid is used as a vector to transport fragmented DNA into the host's cell. Once the vector is inside the host cell, DNA is translocated into the nucleus of the host cells **(Figure 6)**. A mammalian promoter, also placed in the vector, is triggered and initiates transcription of the DNA using the host cell molecular machinery. It helps in the production of harmless spike proteins tagged with a major histocompatibility complex (MHC) to avoid degradation. The harmless spike proteins are recognized by the antigen-presenting cells (APCs) towards the development of specific immune responses and Abs. The usage of this vector in DNA vaccines provide more benefits in comparison to the RNA-based vaccine.

For example, RNA is extremely fragile and must be kept at least -20 degrees Celsius while DNA is quite stable even at room temperature. It also eases the distribution of the DNA vaccine to remote areas. Among DNA vaccines, a current vaccine of interest is Zycov-D. Zycov-D is India's as well as the world's first official DNA-based vaccine. This vaccine needs to be given in 3 separate doses 95. Unlike other vaccines, this vaccine is given via a disposable needle-free injector. It helps in penetrating the skin using a narrow stream. Once DNA is entered the cells, body begins creating its own spike proteins and thus necessary immune response to fend off the SARS-CoV-2.

### 4.3. Protein-based vaccines

Unlike DNA or RNA-based vaccines, protein-based vaccines take a different approach to tackle SARS-CoV-2. In a protein-based vaccine, a specific component of SARS-CoV-2 is isolated and manufactured in a laboratory setting. The spike protein is isolated and placed in a carrier which is injected into the body. The immune response recognizes the spike proteins as foreign and harmful material and begins attacking it **(Figure 6)**. The spike proteins aren't connected to the SARS-CoV-2 virus; thus, there is no risk of infection This type of vaccine doesn't deal with live components, thus making it very safe for people with compromised immune systems 96. The vaccine itself is also very stable and comes from well-researched and well-established technology.

Novavax Inc. created one of the first protein-based vaccines in the US, known as NVX-CoV2373. NVX-CoV2373 is said to be 96% effective against the original strain 97. As more and more variants emerged, Novavax has also come up with a 6-month booster shot. It has proven to aid the Abs against ACE-2 binding inhibition for delta variant. However, the generation of spike proteins is tricky and takes time to identify and determine the best antigen for triggering an appropriate immune response. Further, protein-based vaccines do not last forever, as our body slowly builds immunity also against the carrier.

### 4.4. Viral vector vaccines

The viral vector-based vaccine was another vaccine for actively targeting the COVID-19 pandemic 98. Viral vector vaccines manipulate attenuated viruses wherein whole genetic coding is replaced with the coding of only spike protein. When the engineered virus is injected into the body, appropriate Abs are developed against harmless spike proteins (Figure 5). There are 2 types of viral vectors being used, e.g., replication-defective and replication-competent. In replication-defective vectors, adenovirus vectors carry DNA of the spike protein to the cytoplasm of host cell. Then, DNA makes its way to the nucleus of the host cell, and finally only spike proteins are produced, not the harmful virus. While in the case of replication-competent vectors, a portion of genetic material in the modified virus is replaced by the coding information of the spike protein. Unlike replication-defective vectors, the spike proteins are displayed on the vector itself. Thus, the virus also infects the host cells and makes more spike proteins. However, replication-competent vectors replicate at a lesser strength which allow a more adept immune response.

The Johnson & Johnson/Janssen vaccine showed 72% efficacy against the original strain 99. This efficacy rate is significantly lower than the other RNA-based vaccines. People vaccinated by Janssen have reported headaches, fatigue, muscle aches, nausea, and pain at the injection site. Another concern with the Janssen vaccine is the lack of protection against mild to moderate disease. During the trial phases of the Janssen vaccine, the placebo group had 16 hospitalizations and 19 deaths, while the group with the vaccine had none. This proved that the Janssen vaccine showed 100% efficacy in preventing death/hospitalizations during the trial phase. The latest data shows that Johnson and Johnson is 71% effective against hospitalizations and 95% effective against death caused by the Delta variant.

Although viral vector vaccines are promising, there are many disadvantages as well 100. The viral vector-based vaccines use attenuated viruses; thus, over the time, human body may also create immunity to the vector itself. This immunity could destroy the vector before it has a chance to produce spike proteins. This could alter the efficacy of the vaccine. Another disadvantage is the risk of a completely new infection. Like, in the case of vaccines based on the replication-competent virus, virus may mutate and cause a new infection. Therefore, the body would not only have to fight with harmless spike proteins but also the virus used as a vector 101. In addition to Johnson & Johnson, AstraZeneca has also developed a viral vector vaccine. AstraZeneca has proven to have up to a 70% efficacy rate after the first dose 102. To develop the AstraZeneca vaccine, a modified version of chimpanzee adenovirus, known as ChAdOx1, is used. This adenovirus, like the Johnson & Johnson vaccine, cannot replicate inside the body. Symptoms of the AstraZeneca vaccine include pain at the injection site, headache, and fatigue. Some more serious side effects have been reported, such as thrombosis after receiving either AstraZeneca or Johnson & Johnson vaccine. Roughly 30 out of 5,000,000 people vaccinated with viral vector vaccines reported an adverse event. Those who experienced the side effects were all under the age of 50 and were previously healthy.

### 4.5. Cocktail of vaccines

The more and more variants arise, researchers are exploring new ways to protect the population. One of the methods is the mixing of vaccines 103. Heterologous or mixed vaccination is a technique acquired and approved by the Strategic Advisory Group of Experts (SAGE) and WHO 104. This technique is adopted because of the following reasons. First, if the second dose of the same vaccine is not available, then other types of vaccine can be used. Second, if the first dose of a vaccine does not show the desired results, then the second dose of other vaccines can be taken.

A list of clinical trials and testing of this method can be found here 105,106. A case study showed that currently approved viral-vector-based vaccine AstraZeneca's ChA-dOx1-nCov-19 (ChAd) resulted in thromboembolic events. It left millions of people in dilemma (who took the first dose of the vaccine), whether to take the second dose of the same vaccine or go on with the mRNA-based vaccine. The results of this study showed that the BNT16b2 (Pfizer-BioNTech vaccine) induced significantly higher frequencies of spike-specific CD4+ and CD8+ T cells and, in particular, high titters of neutralizing Abs against B.1.1.7 (Alpha), B.1.351 (Beta), and P.1 (Gamma) variants of SARS-CoV-2 105. So far, no conclusive data is backing up the use of mixing vaccines, but experts say there are no safety concerns regarding the mixing of vaccines. Theoretically, mixing vaccines should be safe!

### 4.6. Innate Immunity

Innate immunity is the first barrier set to protect the body from pathogens. Innate immunity after COVID-19 is important to understand as we still do not fully understand the long-term effects COVID-19, emerging variants, and vaccines.

#### 4.6.1. Neutrophils in SARS-CoV-2 Infection

**Figure 7:**
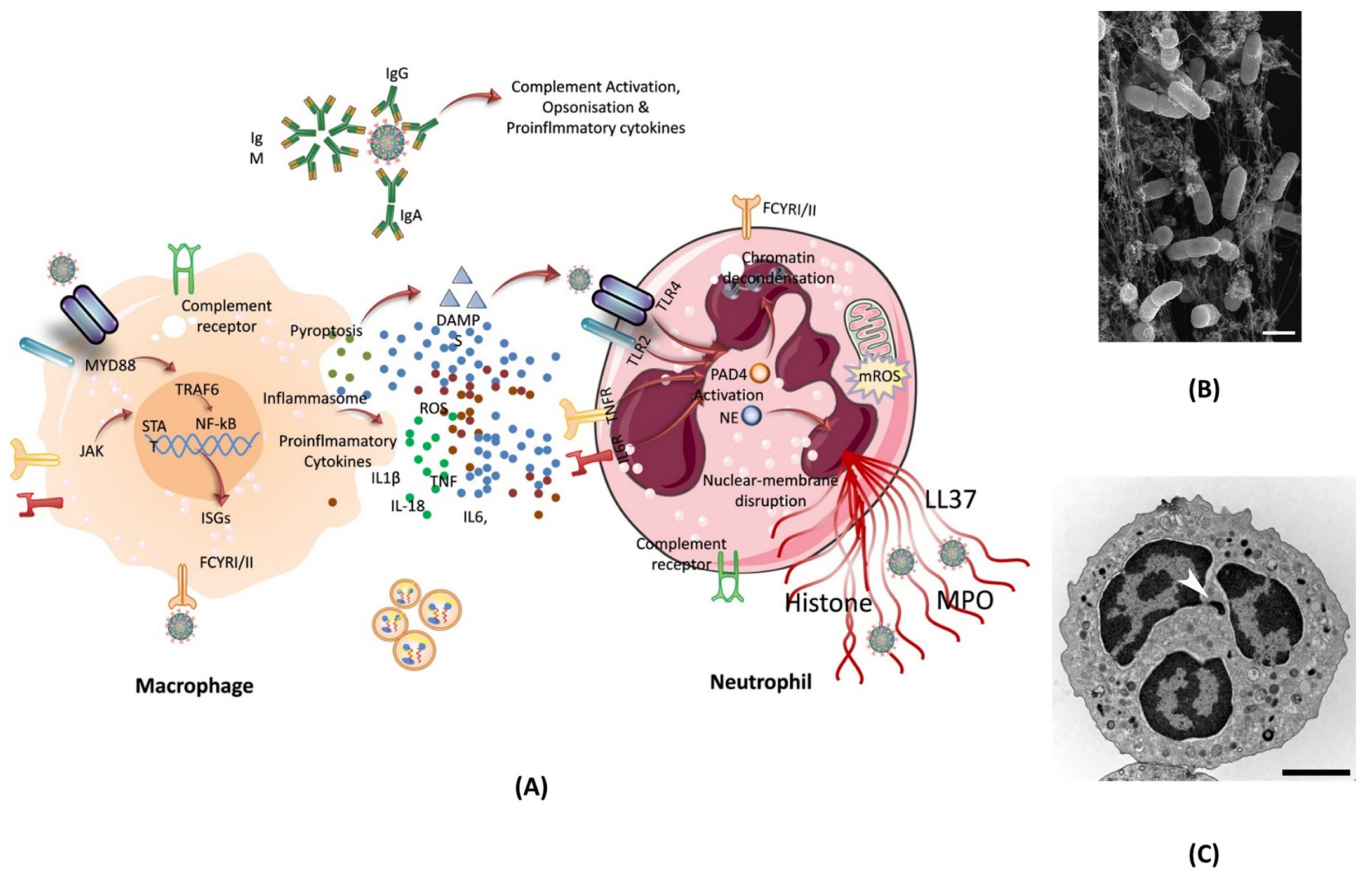
** (A)** The formation of NET by macrophage assisted neutrophil in response to pathogen attack. The reactive oxygen species inside neutrophil activates PAD- 4, and granules release NE which move inside the nucleus to trigger the chromatin decondensation. After decondensation, chromatin expands until it is released from the cell to engulf the pathogen. **(B)** Scanning electron microscopy of human neutrophils incubated with Salmonella, a bacterium that causes typhoid fever and gastroenteritis; adopted from ref. 111 **(C)** Transmission electron microscopy (TEM) of a naive human neutrophil; adopted from ref. 111. *Abbreviations: NE: Neutrophil elastase, MYD88: Myeloid differentiation primary response 88, JAK: Janus kinase, STAT: signal transducer and activator of transcription, NF-kB: nuclear transcription factor-kappa B, ISGs: interferon stimulated genes, DAMPS: damage-associated molecular patterns, TLR2/4: toll-like Receptor 2/4, IL6R: interleukin 6 Receptor, LL37: antimicrobial peptide, PAD4: peptidylarginine deiminase 4, NE: neutrophil elastase, MPO: myeloperoxidase enzyme.*

One of the ways our body can protect itself from harmful agents is through the use of neutrophils. Neutrophils are an essential part of the immune system and makeup almost 70% of all white blood cells. Neutrophils fight against viruses such as COVID-19 following several ways. Regardless of the virus, once an infection is detected in the body, neutrophils reached to the site of infection, which also helps in identifying the type of infection. In case of SARS-CoV-2 infection, neutrophils have been shown to increase in lungs, blood, and nasopharyngeal epithelium. While neutrophils in these areas of the body are common during infection, in COVID-19 patient's neutrophils also show alterations in gene expression. The single-cell RNA sequencing analysis from the blood of COVID-19 patients showed a change in neutrophil numbers. This test, along with marker-based annotation, also showed the presence of pro-neutrophils and pre-neutrophils (known as pro and pre-NEU) 107. The presence of pre and pro NEU is key to understanding COVID-19. The pre-NEU in combination with CD177, CD24, LCN2, BPI, and OLFM4 proteins have been known to be associated with sepsis in COVID-19 patients. Pro-NEU aid in the formation of Neutrophil Extracellular Traps (NET's) which will be discussed in following section. NETs are one of the body's key lines of defense as they aid in the phagocytosis of harmful foreign agents, as discussed below. Also, the severe COVID-19 patients have been found to be associated with immature neutrophils 108. NETs are naturally found in the body's immune system and are extremely important. NETs are bundles of cells called neutrophils and DNA that bind and trap large pathogens which are unable to be destroyed by phagocytosis. Neutrophils are known for having their cytoplasm packed with granules. These special granules are used to aid phagosomes which engulf, kill, and digest harmful pathogens. These granules can also bind to the plasma membrane of foreign bodies and release antimicrobials. NETs are also released when certain pathways are activated via specific inflammatory stimuli. Once these pathways are triggered, neutrophils begin to produce an enzyme complex known as nicotinamide adenine dinucleotide phosphate (NADPH) oxidase. As a result of NADPH, a neutrophil oxidative burst occurs, which produces a large amount of reactive oxygen species (ROS, superoxide). ROS release causes neutrophil elastase (NE), a key component of NET, to be shot out into the cytoplasm 109. Once in the cytoplasm, PAD-4 and NE move to the nucleus and modify the histones to uncoil the chromatin of cells. It's known that PAD-4 works as a catalyst to turn peptidyl arginine into peptidyl citrulline. The citrulline, then causes the decondensation of chromatin which continues to build pressure in the cell until it is released, resulting in NETs 110. This process is known as NETosis **(Figure 7)**.

In COVID-19 patients, high levels of NETs were found in their plasma. The same NETs were also found in the lungs during autopsies. For this reason, NETs can be positively correlated with the disease severity of COVID-19. Many studies show that the presence of IgG antibodies is enough to provoke the release of NETs 112. In most aspects, NETs are seen as a positive component of the immune system. However, recent studies have shown that NETs don't always aid the host body. In the case of COVID-19, NETs are directly induced and controlled by the virus itself. These NETs, once released, have the ability to promote apoptosis within the lung epithelium. The level(s) of NETs also increases the chance of sepsis. In confocal microscopy analysis, the blood of COVID-19 patients was examined for NETs and compared to that of a control. The results showed that patients with COVID-19 had an increased neutrophil count as well as increased NETs release. This was confirmed by seeing the long-ridged NETs rods under the microscope. The structure of the NETs is important to note here because not only were they present in the blood, but the structure itself differed from the traditional NETs. The NETs found in COVID-19 patients were longer than normal NETs rods. It is also important to note that patients with COVID-19 had reduced levels of cells containing Cl-Amidine. Cl-Amidine is an inhibitor that helps to control the amount of PAD-4 enzyme 113.

#### 4.6.2. T Cell Responses

**Figure 8:**
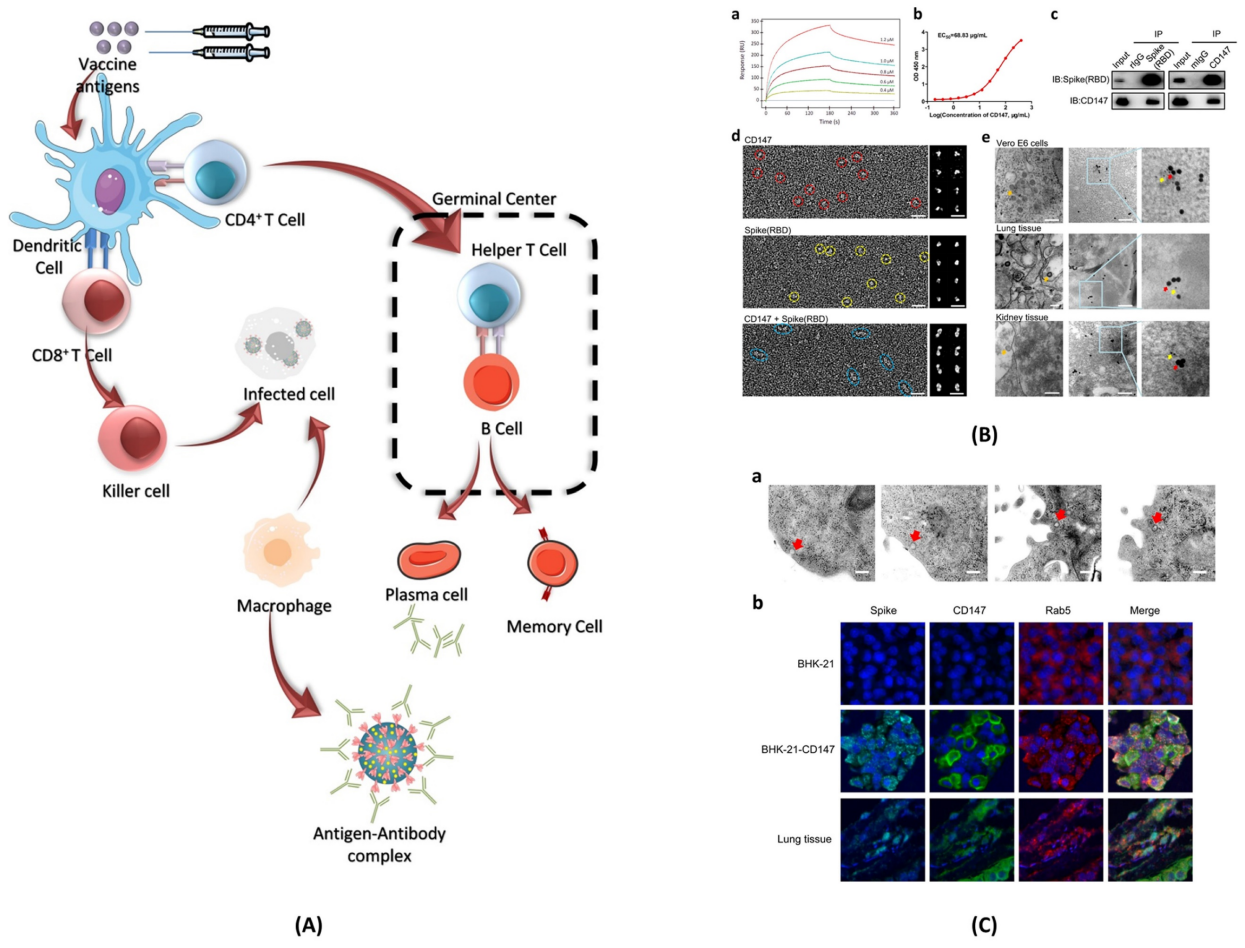
** Immune responses elicited by SARS-CoV-2 mRNA vaccines. (A)** After vaccination, antigen presenting cell activate the CD4+ and CD8+ T cells. CD8+ T cells directly kill the infected cells while CD4+ T cells activate the B cells for the formation of long-lived plasma cells (LLPCs) and memory B Cells (MBCs) inside the germinal center. The macrophages phagocytize and digest the infected cells as well as antigen-antibody complex. **(B)** The interaction of CD147 and spike was detected by SPR assay, ELISA, immune-electron microscope, and Co-IP assay. The mouse IgG and rabbit IgG were served as negative controls; adopted from ref. 117 **(C)** SARS-CoV-2 enters the host cells through CD147-mediated endocytosis and the sequential endocytosis of SARS-CoV-2 was observed in Vero E6 cells by electron microscope. And the co-localization of spike protein, CD147, and Rab5 were analysed in BHK-21-CD147 cells and lung tissues; adopted from ref. 117.

When SARS-CoV-2 enters the body, it infects cells and begins to replicate inside. Host cells release damage-associated molecular patterns (DAMPs), which serve as triggers for the immune system **(Figure 8)**. Macrophages recognize these signals and release chemokines and pro-inflammatory cytokines at the site. Chemokines signal the recruitment of monocytes and T cells to the affected area. T cells engage with CD8 and CD4 receptors to locate peptides displayed on the surface of host cells. These peptides act as signals indicating the presence of the virus taking over the cell. Once T cells identify the peptide, they initiate the destruction of the infected cell by inducing apoptosis.

In regard to COVID-19, the effect of T cells trends to vary as per several studies. It takes about 7-10 days to develop T cell immunity and recover from an infection. Some studies have shown that patients with COVID-19 see a decrease in total amounts of T cells counts during the acute phase of the virus. Studies have not outlined the exact time needed for the acute phase, but it has been shown that the overall amount of T cells decreases over time. As the T cells level decreases, few T cells that remain have an increase in activation. Several data insinuate that T cells found in COVID-19 patients are overworking to compensate for the loss in quantity. The lower the amount of T cells, the harder the remaining T cells must have to work. This also pushes the idea that T cells may be undergoing exhaustion. Once affected by exhaustion, the remaining T cells may start showing poor function, inhibitory receptors, and poor transcriptional regulation of T cells. T cells count in COVID-19 patients also seems to act to tell disease severity. The studies have shown that certain markers of activated T cells such as Tim-3 and PD-1 can signal increased severity of COVID-19 114,115.

#### 4.6.3. Cytokine Storm

COVID-19 has many symptoms, such as loss of smell and fever. While these symptoms are worrisome, some other side effects of COVID-19 are much more severe, i.e., cytokine storms in many patients. A cytokine storm is the overexpression of cytokines in the body due to infection, which may do more harm than good, like multiple organ failure. The over release of cytokines is triggered by numerous factors such as immune evasion, anti-Fc Abs driven cell lysis, cell pyroptosis, etc. As SARS-CoV-2 continues to replicate and mutate, these factors are constantly being triggered and over-releasing cytokines in the body 116.

### 4.7. Vaccine-induced immunity

Although vaccine cannot prevent the virus itself, it has proven to decrease the severity of hospitalizations 77. A COVID-19 vaccine evokes adaptive immunity that include B and T cell responses which can last for a longer duration of time **(Figure 8)**.

The B cells play a key role in immunological response against any pathogen as they mediate antibody-dependent protection. While T cells modulate the B cell activity and contribute directly in the elimination of pathogen-infected cells 118. The long-lasting Abs against viral infections are generated in germinal centres (GCs). Inside the GCs, antigen activated B cells undergo random mutations and change their immunological genes to generate Abs with high affinity for the pathogens 107. The B cells with high affinity are selected and saved from apoptosis, leading to the formation of long-lived plasma cells (LLPCs) and memory B cells (MBCs). LLPCs release Abs, which also include nAbs 108. LLPCs can survive for decades, continuously secreting Abs without any further antigen stimulations 107. The MBCs produced can get activated at the frequent interaction with the viral infection and can give rise to rapid Abs secretion 108. Some of the immune response cases that have been encountered in phase I, II, and III clinical trials of the COVID-19 vaccine are discussed as follows.

#### 4.7.1. Antibody response

The work done by Dorottya Laczko et al. showed that a single dose of 30 µg mRNA vaccine (encoded with the full-length ∆furin S or RBD of SARS-CoV-2) was able to produce strong SARS-CoV-2-binding immunoglobulin G (IgG) titers in mice within a time span of 2 weeks post-immunization 119. The studies have also shown that the Abs response induced by the mRNA vaccines was able to neutralize the virus in vitro (measured by both pseudovirus and SARS-CoV-2 based neutralization assays). Also, nAbs level were sustained for two months or more post-immunization 118. Another interesting observation can be seen from these studies that Abs produced by these mRNA vaccines are dose-dependent, which means a 2nd dose is necessary to induce booster immunization. The antigen specificity plays a key role in the effectiveness of these vaccines 120. It is observed that SARS-CoV-2 S1 mRNA vaccine is not as effective at inducing SARS-CoV-2-binding Abs and nAbs as the RBD mRNA vaccines.

Also, a study was conducted by Parry H and team on 299 patients with B-cell chronic leukemia (CLL) 121. Two types of vaccines were used for vaccination, 154 patients received the BNT162b2 mRNA vaccine, and the other 145 patients received ChAdOx1. Out of the total 299 patients, 286 patients were vaccinated with an extended interval of 10-12 weeks between the first and second dose, while 13 patients were vaccinated as per the standard time period of 3 weeks between first and second dose. The antibody response after COVID-19 vaccination was reduced in patients with CLL, or IgA-deficient or patients on BTKi (Bruton Tyrosine Kinase Inhibitors) therapy.

During the early phases of clinical trials, pregnant women were excluded from vaccination. The understanding of how the maternal Abs would react with the external vaccine is important to avoid any further side-effects in the future. It is also important to know what happens in case a pregnant woman gets infected in her maternal period. Does the viral infection gets transferred to the neonates? To answer all these questions, a study was conducted on 122 pregnant women with cord blood available at the time of birth 122. 85 women received the Pfizer-BioNTech vaccine, and 37 women received the Moderna vaccine. The result showed that eighty-seven pregnant women, tested during the birth, produced an IgG response, 19 women produced both IgM and IgG response, and 16 women had no detectable antibody response 61. This study shows that mRNA-based COVID-19 vaccines in pregnant women lead to maternal antibody production as early as 5 days after the first vaccination dose and transplacental transfer of passive immunity to the neonate as early as 16 days after the first vaccination dose.

Another study found detectable Abs response after mRNA-based COVID-19 vaccination in residential older adults 123. A group of 70 individuals received 2 rounds of vaccination and were found to have Abs with wide variations in relative levels. The response of Abs was lower in advanced age males and steroid users. Recent data also suggest that an in-adequate Abs response is observed after vaccination in patients suffering with haematological malignancies 124.

#### 4.7.2. T cell response

**Figure 9:**
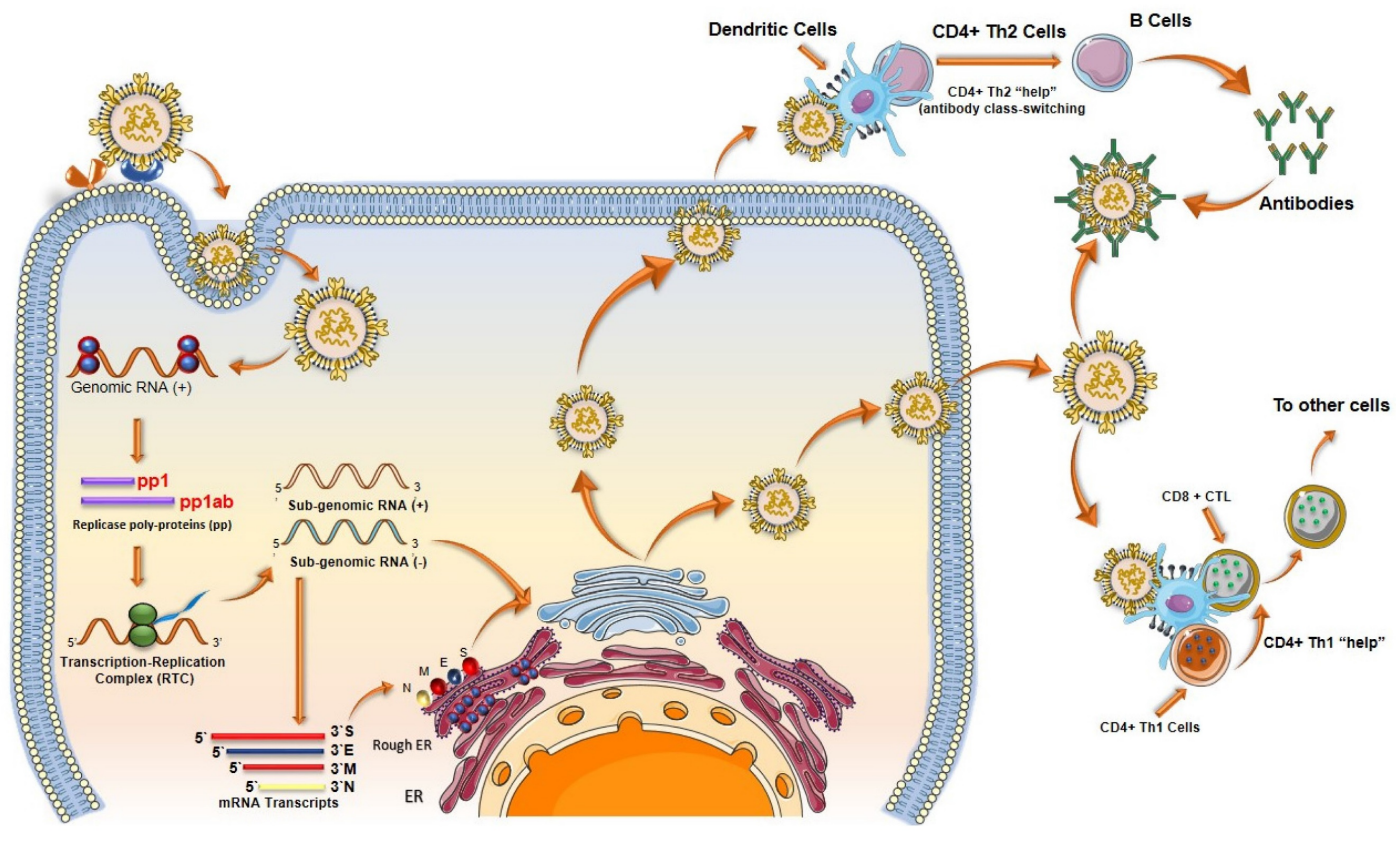
Schematic showing the T cell response after SARS-CoV-2 mRNA vaccines. T lymphocytes attach to antigens presented by dendritic cells and develop into regulatory suppressor, helper, or cytotoxic T lymphocytes. A vital part of the production of Abs and memory B cells, activated helper T cells display receptors on their surface that are unique to vaccine strains.

After the presentation of antigens by dendritic cells, T cells bind with it and proliferate into cytotoxic T cells, regulatory suppressor T cells, or helper T cells. Activated helper T cells express receptors specific to vaccine strands on their surface and play a major role in the generation of Abs and memory B cells (**Figure 9**). When an activated T cell binds with a B cell, it starts to produce lymphokines which have several effects. It helps in the proliferation of activated B cells, which differentiate on either memory B cells or plasma cells. Plasma cells produce Abs specific to the virus contained in the vaccine, whereas memory B cells aid in the future immune response when exposed to an active virus.

The vaccine-induced immunity largely depends on the Ab responses; however, T cell response also plays a crucial role in effective vaccination results. Some of the important benefits of positive T cell response after vaccination can be summarized as follow: among the CD4+ T cells, T follicular helper (Tfh) cells play an important role in regulating germinal center (GC) and affinity-matured Ab responses 118,125. CD4+ T cells also help in inducing optimal CD8+ T cells response. Cytotoxic CD8+ T cells serve as the last resort of body defence if somehow both Abs and B cells fail to react with the pathogen. Cytotoxic CD8+ T cells are tasked with a direct head-to-head encounter with the viral infection by releasing molecules such as granzyme and perforin. The mRNA-based vaccines such as Pfizer-BioNTech, Moderna, and viral vector-based AZD1222, showed a positive T cell response.

#### 4.7.3. Resident memory T cells

Tissue-resident memory T cells (TRM) or simply memory T cells are the subsets of T lymphocytes. Memory T cells are generally found in the localized mucosal and non-mucosal tissues and are activated for rapid response on the second encounter with the pathogen. TRM also recruits other immune cells to the site of infection, initiating multiple counters for the defense against the pathogen.

Most of the vaccines focus on inducing humoral activity, specifically nAbs. However, it has been recognized that other immunological defence mediators also play an important role and should be induced in future vaccines 126,127. The major features of resident memory cells include T cell immunity that can target more conserved regions of the virus, which are often not accessible to Abs. The reactivation of the T cells in the tissue upon re-infection can induce a rapid activation and recruitment of other immunological cells such as B lymphocytes which ensures a total and robust protection against the virus. It is expected that the individuals who are naturally infected with the SARS-CoV-2 virus keep an immunological memory against the virus, and just after the first dose shows a significant spike in the nAbs 126-128. A similar study was done on a number of older patients (above 60 years of age), and the result was the same 129,130. Therefore, we can conclude that the memory of the infection is well preserved.

#### 4.7.4. Immune response relation to dose apart time

It has been reported that a significantly larger amount of Ab titers and boosted T cell response are produced in the individuals when there is an extended gap between the first and second dose of the adenovirus-based COVID-19 vaccine 90,131. The volunteer in this study belongs to the age group of 18 to 55 years who were enrolled in a phase I/II or phase II/III clinical trial of ChAdOx1 nCoV-19. The same study also showed that a third dose (after 28 days of the second dose) of the vaccine could further elevate the amount of Ab titers and T cell responses.

#### 4.7.5. Vaccine induced side-effects

Most of the vaccines currently available on the market are based on either mRNA or viral vector technology. Consequently, many individuals have expressed hesitation about getting vaccinated, primarily due to reported side effects from the doses. Commonly reported side effects include headache, fatigue, myalgia, fever, pain, and redness at the injection site 101,132. Additionally, some rare adverse events have been reported, such as allergic and anaphylactic reactions following mRNA vaccination, as well as thrombosis and thrombocytopenia after non-replicating viral vector vaccination 133-135. Other uncommon side effects observed post-vaccination include myocarditis, Bell's Palsy, Transient Myelitis, Guillain-Barre syndrome, recurrences of herpes-zoster, autoimmunity flares, epilepsy, and orthostatic tachycardia 136. It's important to note that the majority of these adverse events are classified as mild to moderate in severity, emphasizing the overall safety of COVID-19 vaccines.

## 5. Conclusion and future direction

In the unprecedented battle against COVID-19, vaccines have proven pivotal in saving lives and mitigating its severity. Yet, we face additional challenges beyond vaccination. Co-infections with other respiratory illnesses present a perplexing concern, especially for those with pre-existing respiratory conditions. Tailored strategies are essential to effectively manage these co-infections, highlighting the need for a holistic approach beyond vaccines.

As the pandemic's aftermath unravels, we confront a landscape of enduring health challenges in the form of post-COVID-19 conditions. From fatigue to neuropsychiatric issues, these persistent symptoms necessitate multidisciplinary rehabilitation programs and ongoing research. It's clear that our fight against COVID-19 is multifaceted, requiring a deeper understanding of co-infections along with helping people grappling with post-COVID-19 issues. Moving forward, concerted efforts in research, healthcare, and public health will be crucial in navigating the intricate aftermath of COVID-19 and preparing for future health crises.

## Figures and Tables

**Table 1:** Studies conducted in different countries to identify prevalence of COVID-19

Country	No. of studied individuals	Percentage of COVID-19 symptoms	Duration after which COVID-19 symptoms observed	Symptoms	References
Italy	179	87.4%	2 months	Fatigue, dyspnoea, joint pain, chest pain	Carfì et al. (11)
USA	1567	33%	14-21 days	Fatigue, cough, and body aches	Davis et al. (12)
USA	292	35%	2 to 3 weeks	Fatigue, cough, shortness of breath	Tenforde et al. (13)
China	1733	76%	6 months	Fatigue, sleep difficulties, anxiety	Huang et al. (8)
Denmark	9819	34%	60 days	Fatigue, loss of taste & smell, difficulty in attention	Bliddal et al. (14)

**Table 2:**
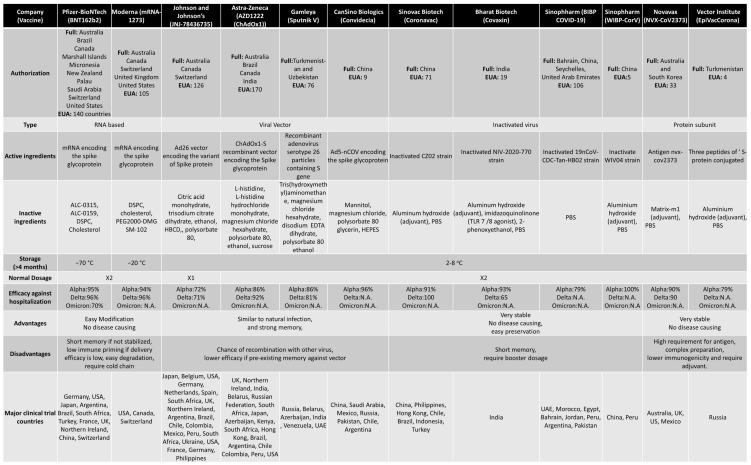
Comparison between different types of EUA approved vaccines.

Abbreviations: ALC-0315, ([(4-hydroxybutyl)azanediyl]di(hexane-6,1-diyl) bis(2-hexyldecanoate)); ALC-0159, 2[(polyethylene glycol)-2000]-N,N-ditetradecylacetamide; DSPC, distearoylphosphatidylcholine; PEG2000-DMG, poly(ethylene glycol)2000-dimyristoylglycerol; SM-102, 8-[(2-hydroxyethyl)[6-oxo-6-(undecyloxy)hexyl]amino]-octanoic acid, 1-octylnonyl ester; HBCD, 2-Hydroxypropyl-β-cyclodextrin; EDTA, Ethylenediaminetetraacetic acid; HEPES, (4-(2-hydroxyethyl)-1-piperazineethanesulfonic acid); PBS, phosphate buffered saline; TLR, Toll-like receptor; N.A., Not available
